# Timing Is Everything

**DOI:** 10.1128/mBio.02140-17

**Published:** 2018-01-02

**Authors:** Luis M. Schang

**Affiliations:** a Baker Institute for Animal Health, Cornell University, Ithaca, New York, USA

**Keywords:** dynamic proteomics, single-cell analyses, cell cycle, gene expression, herpes simplex virus

## Abstract

N. Drayman et al. in their recent article (mBio 8:e01612-17, 2017, https://doi.org/10.1128/mBio.01612-17) have used dynamic proteomics and machine learning to show that the cell cycle state of any individual cell affects the outcome of a productive herpes simplex virus 1 (HSV-1) infection. Cells infected from early G_1_ through S were most permissive for expression of genes from the HSV-1 genome, whereas cells infected in late G_2_ to mitosis were much less so. Most of the infected cells that underwent mitosis became permanently nonpermissive for HSV-1 gene expression afterward. The cell cycle stage accounted for 60% of the success of infection, and cell density and motility accounted for most of the rest. To successfully reactivate, HSV-1 must express its genes in neurons and cells of the spinosum and granulosum epidermis strata. These cells are permanently in the cell cycle stages most permissive for HSV-1 gene expression, and none reenters mitosis, thus maximizing the efficiency of a successful HSV-1 reactivation before the adaptive immunity can control it.

## COMMENTARY

Virology is driven by large numbers. Very large numbers. An event with a one-in-a-million chance is extremely unlikely to occur in a person’s day-to-day life, but it will occur up to 1 to 10 million times among the astonishing number of virions harbored by an infected person or animal. Even relatively rare events are therefore critical in viral pathogenesis, when so many virions infect so many cells. The differences between virions have been addressed multiple times, but those between individually infected cells have been more difficult to tackle (1). There is a generalized long-held view that the individual state of each specific cell is critical to the outcome of an infection with herpes simplex 1 virus 1 (HSV-1), but this view has remained difficult to challenge experimentally. Most of the previous attempts to test it have been limited by the readouts, which have often required high multiplicities of infection, multiple rounds of replication, or estimating the state of the infection by the localization of an infected cell in an infectious focus. Additionally, various analytic methodologies have led to somewhat diverse conclusions, demonstrating the difficulty in studying very rare events.

In a recent article, Drayman et al. (2) have experimentally tested whether the state of the individual cell plays a significant role in determining the outcome of an HSV-1 infection. They first screened a library of cell clones, each expressing a different green fluorescent protein (GFP)-tagged full-length protein from its endogenous locus, by tracking the progression of the infection using a reporter cyan fluorescent protein (CFP) expressed from the HSV-1 genome. The behavior of only 1% of the 400 proteins evaluated was different in productively infected cells. Two proteins stood out: RFX7 and geminin. At the time of infection, both were expressed to their lowest levels in the cells that went on to support the highest levels of HSV-1 gene expression. The levels of these two proteins are directly related to the cell cycle; they are both expressed at their lowest levels immediately after mitosis and at their highest levels at mitosis. These results were thus most consistent with some previous models which had proposed that the cell cycle stage of the infected cells was important for the establishment of a productive HSV-1 infection (3).

Using machine learning to detect the time from last mitosis, Drayman and colleagues directly tested whether the cell cycle stage at which a cell is infected determines the outcome of the infection. They evaluated the levels of expression of CFP from the HSV-1 genome in cells infected at different times after mitosis. HSV-1 gene expression was high in cells infected anytime from immediately after mitosis until approximately 14 h later and then decreased in cells infected until 22 h after the previous mitosis, suggesting that cells are broadly permissive until late G_2_. Similar results, with an even deeper decrease at G_2_/M, were observed by time-lapse live microscopy of infected cells. HSV-1 gene expression was also the highest when cells were infected immediately after release from a thymidine block, when 70% of the cells were in S phase, and lowest when cells were infected 4 h after the release, when 75% were in late G_2_ or mitosis.

Drayman and their colleagues show little to no further viral gene expression in the vast majority of the cells that underwent mitosis after infection. The two most likely mechanisms for this inhibition involve chromatin silencing and failure to migrate to the daughter cells’ nuclei. Cellular chromatin is silenced during mitosis, and HSV-1 chromatin regulates viral gene expression (4–11). HSV-1 genomes may thus well also be silenced in non-transcriptionally competent chromatin during mitosis. Moreover, the HSV-1 genomes have no means to attach to the segregating chromosomes during the mitotic migration and are thus likely not to be incorporated into the daughter cells’ nuclei. Approximately 30% of the infected cells that underwent mitosis reexpressed CFP, suggesting that on average half of the mitosis resulted in one of the two daughter cells further supporting replication after mitosis.

Some key regulators of cell cycle progression had been shown before to be important for HSV-1 gene expression and replication, and the cell cycle state of the infected cell had been directly implicated in the success of a productive infection. A variety of cyclins and cyclin-dependent kinases (CDKs) have been shown to be altered during, or important for, HSV-1 replication or explant-induced reactivation (12–18). Inhibitors of several of the cell cycle-promoting cyclin-dependent kinases (CDKs) inhibit HSV-1 transcription and replication (19–21) and may even inhibit HSV-1 encephalitis (22). HSV-1 ICP0 was shown to inhibit cell cycle progression at G_1_/S and G_2_/M (23). ICP0 mutants were shown to plaque more efficiently in cells that were reentering the cell cycle, although this difference was later attributed to cellular stresses, not the particular cell cycle stage of the infected cells (3, 24–26). These experiments used plaque formation to assess the efficiency of the infection in the first infected cell (3, 24, 26). This readout requires replication of the virus in multiple cells that are no longer synchronized after the first 24 h or so. It is conceivable, almost expected, that the 2- to 3-fold differences observed in individual cells in the experiments now reported would have been diluted in the subsequent rounds of infection of nonsynchronized cells. In these previous experiments, moreover, the cells had to be synchronized before infection, an approach that precludes analyzing whether the initial cell cycle state of undisturbed cells is a determinant of the success of infection.

While the focus of the current study is on productive HSV-1 infections, HSV-1 preferentially establishes nonproductive (latent) infections in neurons. Neurons are arrested in a G0/G_1_-like state and never reach G_2_ or mitosis. It is thus intrinsically obvious that cellular factors other than the stage of the cell cycle also play a most determinant role in the outcome of the infection. Moreover, classic experiments by Cohen et al. (27) had shown already in 1971 that the success of infections at high multiplicities (200 HSV-1 virions per cell) is not significantly different in cells at different stages of the cell cycle. It is thus equally clear that the restriction in cells infected at different stages of the cell cycle is not absolute and can be overcome, given for example a sufficiently high multiplicity of infection.

**FIG 1:**
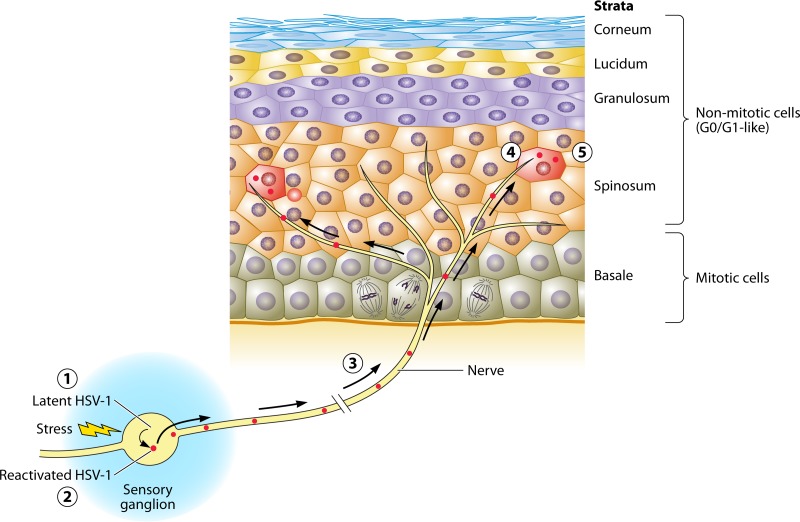
HSV-1 establishes latent infections in neurons in the sensory ganglia, which are permanently arrested in a G0/G_1_-like state and will never reenter mitosis (1). Therefore, reactivation occurs in cells that are in a most permissive state for viral gene expression (2). The reactivated virions then travel in the axons (3) to their termini on the epidermis (4), where they are most likely to be transmitted first to cells in the spinosum and granulosum strata (5). These cells are also in the most permissive states and do not reenter mitosis. The reactivated virus then replicates in these cells and spreads to the neighboring ones, completing a successful reactivation. The entire HSV-1 reactivation process thus occurs in cells that are most permissive for viral gene expression, maximizing the efficiency of the reactivation process before the adaptive immunity can control it.

This work has important implications in our understanding of the biology of HSV-1. Together with the density of cells around the infected one and the mobility of the infected cells, the stage of the cell cycle predicted approximately 60% of the success of HSV-1 in establishing a productive infection. The density of cells can be presumed to be reasonably homogeneous in human skin and mucosa, where most of the infected cells are nonmotile keratinocytes (Fig. 1). One could presume that the particular cell cycle stage of the infected cell in a patient is thus a critical determinant of the probabilities of success of the infection. This restriction would probably be less critical in the primary infection in immunologically naive individuals, in whom only the innate immune responses must be overcome. The infecting virions likely have a reasonable time window to establish a productive infection, even if the success rate of each infection is rather low, before the development of the specific immune responses. Reactivation of HSV-1 occurs in hosts which have already developed mature adaptive immune responses. In this context, it may well be critical for HSV-1 to promptly replicate and be shed before the immune system controls the reactivation episode. Reactivation occurs in peripheral sensory neurons, which, like all neurons, are in a G0/G_1_-like state. G_1_ is described in the work of Drayman et al. as highly permissive for viral gene expression. The cells most likely to be first infected after reactivation are those in direct contact with the nerve termini at the spinosum or granulosum stratum of the epidermis (Fig. 1). These cells do not enter mitosis ever again and are thus permanently in a state described in the work of Drayman et al. as most permissive for viral gene expression and replication. The entire reactivation process thus occurs in cells that are in the most permissive states for viral gene expression (Fig. 1), maximizing the efficiency of the process to ensure the production of infectious virions before the immune system can control the reactivation.

Perhaps the most surprising result of the reported experiments is that the efficiency of gene expression from the viral genomes did not change much between G_1_ and S. Only progression into G_2_/M resulted in major inhibition of viral gene expression. Equally surprising is that 14% of infected cells still progressed into mitosis. Cellular DNA synthesis is inhibited in infected cells (28), although it is fascinatingly stimulated in neighboring noninfected ones (29), which is by definition an inhibition of progression through S (defined as the DNA synthesis phase). The molecular analyses result in a more complex picture, however, as infected cells express proteins, complexes, or binding activities that in noninfected cells are characteristic of different stages of the cell cycle (30–34), indicating more of a general dysregulation than a specific block. Nonetheless, the consensus is interpreted as meaning that HSV-1 inhibits cell cycle progression at very late G_1_ or G_1_/S, or early S, with another blockage at G_2_/M. These blocks have been often considered to be important for HSV-1 infection. However, HSV-1 gene expression is now shown to still be high in cells in S phase, after release from a thymidine block, and the efficiency of infection did not change significantly before late G_2_/M. The observed effects of infection on the cell cycle may thus not be as critical as once thought. In support of this model, there is no known HSV-1 mutant that fails to replicate as a consequence of a failure to inhibit cell cycle progression, whereas several mutants have replicative defects in arrested cells. The clearer understanding of the relationships between HSV-1 replication and the cell cycle coming from the current experiments should come as good news in the development of HSV-1 mutants as oncolytic agents, development which requires viral mutants that most usually depend on the cell cycle progression by the cancerous cells to be killed.

In conclusion, the recent article by Drayman et al. provides direct experimental evidence that the cell cycle stage of a cell plays a major, albeit not exclusive, role in determining the outcome of a productive HSV-1 infection and is most likely an important factor in the pathobiology of HSV-1 infections.
